# Spatial heterogeneity and temporal evolution of malaria transmission risk in Dakar, Senegal, according to remotely sensed environmental data

**DOI:** 10.1186/1475-2875-9-252

**Published:** 2010-09-03

**Authors:** Vanessa Machault, Cécile Vignolles, Frédéric Pagès, Libasse Gadiaga, Abdoulaye Gaye, Cheikh Sokhna, Jean-François Trape, Jean-Pierre Lacaux, Christophe Rogier

**Affiliations:** 1 Unité de recherche en biologie et épidémiologie parasitaires, Equipe 7 "Maladies émergentes et moustiques"/Unité de Recherche sur les Maladies Infectieuses et Tropicales Emergentes - URMITE - UMR6236, Institut de Recherche Biomédicale des Armées, Allée du Médecin colonel Jamot, Parc du Pharo, BP60109, 13262 Marseille cedex 07, France; 2 Unité d'entomologie médicale, Equipe 7 "Maladies émergentes et moustiques"/Unité de Recherche sur les Maladies Infectieuses et Tropicales Emergentes - URMITE - UMR6236, Institut de Recherche Biomédicale des Armées, Allée du Médecin colonel Jamot, Parc du Pharo, BP60109, 13262 Marseille cedex 07, France; 3 Centre National d'Etudes Spatiales - Service Applications et Valorisation - 18 avenue Edouard Belin, 31401 Toulouse Cedex 9, France; 4 Observatoire Midi-Pyrénées, Université Paul Sabatier, 14 avenue Edouard Belin, 31400 Toulouse, France; 5 Institut de Recherche pour le Développement, UMR198 "Paludisme et maladies émergentes en Afrique de l'Ouest: détection, épidémiologie et lutte"/Unité de Recherche sur les Maladies Infectieuses et Tropicales Emergentes - URMITE, Route des Pères Maristes, BP 1386, 18524 Dakar, Sénégal

## Abstract

**Background:**

The United Nations forecasts that by 2050, more than 60% of the African population will live in cities. Thus, urban malaria is considered an important emerging health problem in that continent. Remote sensing (RS) and geographic information systems (GIS) are useful tools for addressing the challenge of assessing, understanding and spatially focusing malaria control activities. The objectives of the present study were to use high spatial resolution SPOT (*Satellite Pour l'Observation de la Terre*) satellite images to identify some urban environmental factors in Dakar associated with *Anopheles arabiensis *densities, to assess the persistence of these associations and to describe spatial changes in at-risk environments using a decadal time scale.

**Methods:**

Two SPOT images from the 1996 and 2007 rainy seasons in Dakar were processed to extract environmental factors, using supervised classification of land use and land cover, and a calculation of NDVI (Normalized Difference Vegetation Index) and distance to vegetation. Linear regressions were fitted to identify the ecological factors associated with *An. arabiensis *aggressiveness measured in 1994-97 in the South and centre districts of Dakar. Risk maps for populated areas were computed and compared for 1996 and 2007 using the results of the statistical models.

**Results:**

Almost 60% of the variability in anopheline aggressiveness measured in 1994-97 was explained with only one variable: the built-up area in a 300-m radius buffer around the catching points. This association remained stable between 1996 and 2007. Risk maps were drawn by inverting the statistical association. The total increase of the built-up areas in Dakar was about 30% between 1996 and 2007. In proportion to the total population of the city, the population at high risk for malaria fell from 32% to 20%, whereas the low-risk population rose from 29 to 41%.

**Conclusions:**

Environmental data retrieved from high spatial resolution SPOT satellite images were associated with *An. arabiensis *densities in Dakar urban setting, which allowed to generate malaria transmission risk maps. The evolution of the risk was quantified, and the results indicated there are benefits of urbanization in Dakar, since the proportion of the low risk population increased while urbanization progressed.

## Background

### Malaria and urbanization

Urbanization is occurring at a rapid pace in Africa, and the United Nations forecasts that by 2050, more than 60% of the African population will live in cities [1]. Inescapably, those changes will have consequences on the health of local populations. Regarding malaria, many papers and reviews have reported the existence of transmission in urban areas, even if levels are usually lower than in peri-urban and rural places [2,3]. The epidemiology of malaria in cities is specific, and the urban form of the disease is considered to be an emerging health problem of major importance in Africa [4].

The urban malaria burden, as well as its spatial and temporal distribution, is closely related to a wide range of factors, such as the degree and type of urbanization, the density of the human population, vector control measures, access to health care [2,5] and adaptation of the vector to new or polluted breeding sites [6-9]. Urbanization has a great impact on the composition of the vector system and malaria transmission dynamics [10]. Moreover, in urban settings, blood meal sources are abundant, dispersion of the vectors is low and malaria transmission is highly driven by the proximity of breeding sites [11,12]. Malaria risk is heterogeneous over small distances, and transmission can vary among different districts of the same city, as shown in Brazzaville [13] and Dakar [14].

### Malaria and remote sensing

Risk maps can be useful for decision-makers who are seeking to address the challenge of assessing, understanding and spatially focusing malaria control activities [15]. Over the last several decades, remote sensing (RS) and geographic information systems (GIS) have become popular tools for evaluating the environmental, meteorological and climatic factors leading to the geographical and temporal distribution of malaria risk [16-20]. The majority of studies have been conducted in rural settings, but some research has focused on cities. For example, previous studies have conducted direct and indirect identification of *Anopheles *breeding sites, sought to predict malariometric indices and assessed impact of environmental changes on malaria risk [21-24].

### Malaria in Dakar

In Dakar, the capital city of Senegal, parasite rates and incidences of clinical malaria attacks in the city and its periphery have been at low levels relative to continent-wide rates [2,11,25,26]. Nevertheless, some malaria cases have been recognized as autochthonous [26], and severe cases have been reported [27-29]. Additionally, placental malaria infections have been associated with preeclampsia in pregnant women with poor malaria immunity [30]. In this clinical context, local malaria transmission has been studied for several decades and has been assessed in Dakar [14,31] and its close suburb Pikine [11,32].

### Objectives

The objectives of the present study were to use high spatial resolution SPOT (*Satellite Pour l'Observation de la Terre*) satellite images to identify some urban environmental factors associated with *Anopheles arabiensis *densities in Dakar, to assess the persistence of these associations and to describe spatial changes in at-risk environments at a decadal time scale.

## Methods

### Study site

Dakar (14°40'20" North, 17°25'22" West), the capital city of Senegal, is located in the Cap-Vert peninsula at the westernmost point of Africa. The altitude peaks at 104 m above sea level (Mamelles). The climate is mild sahelian. The hot and wet season lasts from June to November with average temperatures between 24 and 30°C. The cool and dry season lasts from December to May with average temperatures between 19 and 25°C. The first rains generally occur at the end of June or the beginning of July, and the last ones at the beginning of October. In 1994, 1996 and 2007, the annual rainfalls were 252, 350 [25,26] and 178 mm (data from the national weather forecast), respectively. The estimated population of the Dakar urban area was close to 1 million inhabitants in 2005, accounting for about 20% of the country's population. The population density was about 12,000 inhabitants per km^2^.

### Entomological data from the literature

Part of the entomological data used in the present study were from two studies of Diallo *et al*, which were conducted in both the south [25] and centre [26] sanitary districts of Dakar, and published in 1998 and 2000, respectively.

The first paper described entomological fieldwork from June 1994 to May 1995 in 13 sites, and the second paper reported mosquito prospecting from March 1996 to February 1997 in 12 sites. In both studies, each site was 1.5 km (North-South) and 1 km (East-West) distant from the adjacent ones. Adult mosquito sampling was carried out once every month during the studied period by human landing catch, both indoors (one catching point) and outdoors (one catching point).

In the southern district, a total of 308 person/nights of adult mosquito captures were undertaken. Among the 16 637 females collected, 83 (0.5%) were *Anopheles*, 81 were *An. arabiensis *and two were *Anopheles pharoensis*. *Anopheles arabiensis *densities were low, peaking at 1.76 bites per person per night in October 1994 (mean value for all sites). In the centre district, a total of 308 person/nights of capture were also conducted. Among 6 157 collected female mosquitoes, 92 (1.5%) were *Anopheles*, and all of them were *An. arabiensis*. Maximum aggressiveness was 2.25 bites per person per night in September 1996 (mean value for all sites). In both districts, none of the *An. arabiensis *were CSP antigen positive.

For the purpose of the present study, the mean number of *An. arabiensis *bites per person per night for both of the 12-month study periods was considered and hereafter is called the "1994-97 aggressiveness". Each site was surveyed between 22 and 24 nights so the full transmission season (September-October) was covered. Thus, even if the study periods for the two sanitary districts were a little bit staggered, it was considered that the figures from the two studies were comparable.

### Field entomological data collection

The field work in the present study has been described elsewhere [14]. Briefly, it was conducted in Dakar and Pikine, a nearby suburb. The studied sites were sampled in order to cover as many diverse environments as possible in terms of type of urbanization and vegetation. The results for nine studied areas are reported in the present paper. Adult mosquito sampling was carried out once every two weeks during September and October 2007. Human landing catch of adult mosquitoes was conducted both indoors (one catching point) and outdoors (two catching points) for a total of four nights of capture in each of the studied areas. Published *An. arabiensis *densities were calculated as the mean aggressiveness for two months of mosquito collection [14]. In order to compare those data with the data from the centre and south districts, aggressiveness values were averaged over a 12-month period for the present study, assuming that most of the annual *Anopheles *density was caught during the September-October period.

### Geolocation of study sites

The geolocations of the catching points of the south and centre districts were not reported in the published articles. Therefore, a member of the team who had participated in the fieldwork in 1994-95 and 1996-97 went back to the capture houses with a GPS (global positioning system) receiver to record appropriate geographic coordinates. Between both districts, 22 sites out of 25 were successfully geolocated on the ground, and three points were approximated. The "Hann - Village" and "Caserne Gendarmerie de Potou" catching houses could not be relocated, so the point was located in the neighbourhood. In "Bop-CerfVolant", the slum existing in 1996-97 was destroyed, so the point was set in the formerly built-up area identified on available aerial photographs from 1997. For the 2007 sites, precise geographic coordinates were available for every capture location. The geographic centre of the three catching points was used for each of the nine sites as the unique location. A description of all the sampled areas is available in the three original articles.

### Satellite images and aerial photographs

SPOT View imagery products were acquired for the following dates: 30 October 1996 and 26 September 2007. Images were "Level 3" pre-processed (orthoimages), so they were already georeferenced, and their map projection (UTM zone 28, WGS 84 datum) was based on ground control points and a digital elevation model (DEM). The location accuracy of the images was less than 10 m [33]. For 1996, the SPOT-4 image had a 20-m spatial resolution and three spectral bands: two in the visible (green and red) and one in the near infrared (NIR). For 2007, one SPOT-5 image had a 2.5-m spatial resolution and the same three spectral bands than the 1996 image. A second SPOT-5 image had one band at a 10-m spatial resolution for short wave infrared (SWIR). These SPOT images covered a large area of the Cap-Vert Peninsula, so views were resized to cover Dakar city and its suburbs until Pikine (lower left corner: 17°31'44"W 14°38'44"N; upper right corner: 17°23'21,95"W 14°47'26,23"N).

A panchromatic QuickBird image (0.61 m resolution, projection UTM zone 28, WGS 84 datum) from 2005 was also available for the studied urban area. Finally, aerial photographs from 1997 were scanned from paper at the "*IGN France International - Bureau de Coordination Projet Cartographie du Sénégal 1/200 000*" in Dakar. Quickbird image and aerial photographs were georeferenced on the SPOT images using control points in ENVI 4.3 (ITTVIS software). QuickBird image and aerial photographs were used for visual support only, whereas SPOT images were processed for the analysis.

### Pre-processing of SPOT images

All image processing was conducted with ENVI 4.3. A common mask of the sea was digitized and applied to all the images, whereas specific cloud masks were digitized for each date. Clouds and their shadows covered 379 Ha on the 2007 image but did not hide any of the entomological sampling zones. No clouds were observed in the studied zone on the 1996 image. To reach the objective of comparing images of different spatial resolutions, the 2007 views were resampled at 20 m, averaging values of the pixels contributing to the output pixel. Both resulting images were stacked to produce a new multiband image encompassing the full spectral resolution, hereafter called the 2007 image. Because comparisons of multi-date images could be impeded by differences in atmospheric conditions from one date to another, internal average relative reflectance (IARR) calibration was undertaken to normalize images to a scene average spectrum [34,35]. All further image processing was based on these calibrated images.

### NDVI and distance to vegetation

The NDVI is the ratio of two spectral bands available in SPOT imagery and is calculated as follow: NDVI = (NIR - Red)/(NIR + Red). The result can range from -1 to +1, where high values correspond to a dense and active vegetation cover. The NDVI images were calculated from both the 1996 and 2007 images. A threshold of 0.1 was used to best separate vegetated from non-vegetated pixels and to produce binary images for 1996 and 2007. This step was conducted by an operator with good knowledge of Dakar city, who was aided by the examination of the QuickBird image and the aerial photographs. To eliminate isolated pixels, a majority filter was applied to the resulting binary images. All pixels were replaced by the majority class in a passing window (62.5 × 62.5 m).

### Land use and land cover classification

Various classification techniques were investigated. Unsupervised classification (ISODATA) was ignored as many confusions occurred, e.g., between water and dark asphalt. Supervised maximum likelihood classification was chosen to generate maps of land use and land cover. Each pixel was assigned to the class having the highest probability to be the correct one based on a set of training areas. No exclusion threshold was defined, so every pixel of the studied zone was classified.

Training polygons were digitized by an operator with good knowledge of the town who photo-interpreted and examined the SPOT and QuickBird images and aerial photographs. Separability of the different classes was regularly computed to assist the definition of the polygons. Training sets were chosen exclusively where no visible land changes occurred between 1996 and 2007. Thus classifications were done in 1996 and 2007 with the same training polygons, which should maximize the comparability of the results. Three-hundred forty-nine training polygons were digitized in the 1996 and 2007 images. They covered 127 ha, representing about 1% of the total zone (excluding the sea). Thirteen land cover classes were defined, which were distributed as five urban classes (depending of the type of buildings and soils), one vegetation class, one water class and six bare soil classes (asphalt, sand, other types of soils, mixed or not with vegetation) (Additional File 1).

The quality of the resulting supervised classified images was assessed by calculating the kappa statistics, which provide a measurement of the agreement between the classes issued from the classification and the training polygons. The majority filter (62.5 × 62.5 m) was also applied to the resulting images to eliminate isolated pixels. The sea coast was masked as the classification quality was low for this particular land cover.

### Geographic information system (GIS)

A GIS was built in ArcGIS 9.2 (Environmental Research Systems Institute, Redlands, CA). The layers were added as follows: map of vegetation (corresponding to the filtered map of NDVI > 0.1) for 1996 and 2007, results of the filtered supervised classifications for 1996 and 2007, 23 points corresponding to the 1994-97 sampling locations with related aggressiveness values and nine points for 2007 also with aggressiveness values. For every catching point, the Euclidian distance to the first vegetated pixel was computed, and the number of pixels of each of the 13 land use and land cover classes at several radius buffers (from 100 m to 500 m) was calculated. Spatial autocorrelations between aggressivenesses were investigated using the Moran's I index.

### Statistical analysis

Statistical associations between the 1994-97 aggressiveness and the data issued from the 1996 image were first investigated to identify which environmental factors were associated with the *An. arabiensis *densities (Step 1). Then, associations between the 1994-97 aggressiveness and the data issued from the 2007 image were examined in order to assess the persistence of the associations over time (Step 2). Finally, external validation (Step 3) was undertaken by researching the associations between the 2007 aggressiveness and the 2007 image, using the variables found to be significantly associated in Step 1. Thus, the quality of the predictions of *An. arabiensis *densities from Step 1 was assessed. The dependent variables were square root transformed, and linear regression models were fitted. All combinations of classes in the 100-m to 500-m radius buffers were tested as independent variables in order to obtain the best association. All statistical analyses were performed using STATA 9.0 (Stata-Corp LP). Spatial autocorrelation was researched among the residuals of the fitted regressions using the Moran's I index in ArcGIS 9.2.

### Risk maps

Following the results of the statistical analysis, risk maps were drawn for 1996 and 2007 by computing for every pixel of the studied zone the environmental factors found to be statistically associated with aggressiveness. This calculation was done for populated areas only. Masks were applied specifically on the 1996 and 2007 images to hide any non-urban pixels, such as vegetation, water, swamp areas and bare soils. The masks were issued from the results of the supervised classification and assisted by a manual digitisation. The areas that were not masked depicted the built-up areas and allowed the urban evolution in the 11 years to be described and quantified.

## Results

### Image processing

All satellite image pre-processing was successfully conducted and enabled the generation of vegetation images as well as land use and land cover maps for 1996 and 2007. The quality of the supervised classification was validated thanks to the high kappa coefficient (0.85 for the 1996 image and 0.95 for the 2007 image). The built-up area was defined as the total surface of all urban classes plus the asphalt class.

### GIS and statistical analysis

All entomological data are presented in the five first columns of Tables 1 and 2 for the 1994-97 and 2007 studies respectively.

**Table 1:** Description of the 1994-97 entomological data and the environmental variables evaluated from 1996 and 2007 SPOT images.

Catching point	N° of catching point	District	Geographic coordinates (Decimal degrees)	*An. * *arabiensis * aggressiveness (number of bites **/person/night,**, averaged for 12 months)	Year*	Built-up area in 300-m radius buffer (Ha)**	Distance to vegetation (NDVI > 0.1) (m)	Risk map class from the final model
Usine Niari Talli	1	Centre	14.7105; -17.4519	0				
					1996	28.1	447	Low
					2007	28.2	449	Low
Point E (Zone B)	2	Centre	14.6976; -17.4558	0				
					1996	26.0	149	Medium
					2007	26.8	189	Low
SICAP Liberte I	3	Centre	14.7083; -17.4605	0				
					1996	25.3	286	Medium
					2007	28.0	409	Low
HLM III	4	Centre	14.7107; -17.4447	0				
					1996	26.4	404	Low
					2007	27.5	449	Low
Derklé- Castor	5	Centre	14.7238; -17.4497	0				
					1996	24.3	351	Medium
					2007	26.5	438	Low
SICAP Liberte VI	6	Centre	14.7244; -17.4630	0				
					1996	16.2	55	High
					2007	26.0	181	Low
Hann Village	7	Centre	14.7177; -17.4365	0.15				
					1996	21.0	167	Medium
					2007	21.5	162	Medium
Cite des eaux	8	Centre	14.7246; -17.4431	0.19				
					1996	20.3	0	Medium
					2007	22.0	98	Medium
Bop - Cerf Volant	9	Centre	14.6992; -17.4498	0.38				
					1996	18.0	117	High
					2007	18.5	25	High
Hann - Pêcheurs B	10	Centre	14.7286; -17.4234	0.46				
					1996	14.5	343	Low
					2007	14.7	316	Low
Hann - Pêcheurs A	11	Centre	14.7192; -17.4320	0.85				
					1996	19.1	55	Medium
					2007	21.0	65	Medium
Zone des hydrocarbures	12	Centre	14.7144; -17.4385	1.5				
					1996	15.4	0	High
					2007	18.8	50	High
Mboth (Plateau)	13	South	14.6701; -17.4357	0				
					1996	28.3	338	Low
					2007	28.0	317	Low
Gueule - Tapée	14	South	14.6802; -17.4572	0				
					1996	28.2	487	Low
					2007	27.6	398	Low
Diecko Nord (Médina)	15	South	14.6752; 17,43237	0				
					1996	28.3	424	Low
					2007	28.2	392	Low
Niayes Thioker	16	South	14.6716; -17.4413	0.04				
					1996	28.2	351	Low
					2007	27.6	251	Low
Fann - Hock	17	South	14.6817; -17.4614	0.04				
					1996	21.0	135	Medium
					2007	21.2	125	Medium
Cite du port autonome (Plateau)	18	South	14.6752; -17.4323	0.04				
					1996	23.4	151	Low
					2007	24.1	295	Low
Camp Dial Diop	19	South	14.6587; -17.4371	0.18				
					1996	13.2	28	High
					2007	18.5	90	High
Caserne des sapeurs pompiers (av Malick Sy)	20	South	14.6816; -17.4381	0.21				
					1996	27.6	476	Low
					2007	27.1	343	Low
HLM Fass	21	South	14.6952; -17.4507	0.21				
					1996	23.4	143	Medium
					2007	19.0	147	High
Caserne gendarmerie de Potou	22	South	14.6947; -17.4346	0.25				
					1996	26.8	120	Low
					2007	26.8	491	Low
Fann hôpital	23	South	14.6900; -17.4663	0.79				
					1996	7.2	7	High
					2007	12.3	0	High

*1996: data extracted from the 1996 SPOT-4 image (20 m) and 2007: data extracted from the 2007 SPOT-5 image (2.5 m degraded to 20 m)

** Total surface in a 300-m radius buffer is 28.3 Ha

The aggressivenesses are given for 23 adult *Anopheles *catching points in the south and centre districts of Dakar. All values are rounded.

**Table 2:** Description of the 2007 entomological data and the environmental variables evaluated from 2007 SPOT image.

Study area	N° of catching point	Geographic coordinates (Decimal degrees)	Total number of *An. arabiensis* (12 person/nights of capture in each study area)	*An. arabiensis *aggressiveness (number of bites/person/night, averaged for 12 months)	Built-up area in 300-m radius buffer (Ha)*	Risk map class from the final model
Pikine	1	-17.4634; 14.6892	180	2.50	15.2	High
Universite	2	-17.4322; 14.7321	138	1.92	10.6	High
Hann Maristes	3	-17.4711; 14.7453	60	0.84	15.0	High
Ouest Foire	4	-17.4443; 14.6849	49	0.68	18.2	High
Gibraltar	5	-17.4353; 14.7159	43	0.60	27.4	Low
Yarakh	6	-17.4600; 14.7238	42	0.58	21.9	Medium
Liberte 5	7	-17.4448; 14.7496	8	0.11	27.8	Low
Grand Medine	8	-17.4803; 14.7609	3	0.04	26.4	Low
Yoff	9	-17.3987; 14.7584	1	0.01	27.1	Low

* Total surface in a 300-m radius buffer is 28.3 Ha

The aggressivenesses are given for nine adult *Anopheles *catching points in Dakar and Pikine. All values are rounded.

Step 1. Processing of ecological and entomological data in the GIS allowed the environmental variables to be extracted from the 1996 image for the 23 *Anopheles *catching points from the 1994-97 studies. Results are presented in Table 1. The Moran's I index was -0.01, indicating that the pattern where neither clustered nor dispersed. The z-score was equal to 0.9 standard deviations so it was associated with a high p-value. Those results were not statistically significant, possibly due to the small number of observations. In the absence of evidence of spatial autocorrelation between the 23 aggressiveness values from 1994-97, no cluster parameter was taken into account in the statistical analyses. In the univariate analyses, the anopheline aggressiveness measured in 1994-97 was significantly negatively associated with the distance to the vegetation (R^2 ^= 0.38) and the built-up area in a 300-m radius buffer (R^2 ^= 0.42) in the 1996 images (Table 3). When excluding one by one the observations for which the geographical locations were approximated, the parameters did not differ from the ones estimated in models including all the observations. Thus, all the observations where kept in the models.

**Table 3:** Environmental factors evaluated from 1996 (Step 1) and 2007 (Step 2) SPOT satellite images and associated with the 1994-97 anopheline aggressiveness.

	1996 SPOT-4 image (20 m) (Step 1)				2007 SPOT-5 image (2.5 m degraded to 20 m) (Step 2)			
	**Coefficient**	**95% IC**	**p-value**	**Adjusted R^2^**	**Coefficient**	**95% IC**	**p-value**	**Adjusted R^2^**

Distance to vegetation (per 100 m and square root transformed)	-0.33	-0.51; -0.15	0.0010	0.38	-0.41	-0.60; -0.21	0.0003	0.45
								
Built-up area in 300-m radius buffer (per Ha)	-0.04	-0.06; -0.02	0.0005	0.42	-0.06	-0.07; -0.04	< 0.0001	0.57

Univariate linear regression. Twenty-three observations. Independent variables are not rounded. Anopheline aggressiveness is quare root transformed.

Step 2. The GIS also allowed the extraction of the environmental variables from the 2007 image for the 23 *Anopheles *catching points from the 1994-97 studies (Table 1). The R^2 ^was 0.45 for the linear regression including the distance to vegetation and the R^2 ^was 0.57 for the linear regression including the built-up area in a 300-m radius buffer (Table 3). The statistical associations found in Step 1 persisted over time. Table 4 contains the statistical parameters for buffers of different sizes and indicates that the 300-m buffer fit the best.

**Table 4:** Built-up area in 100- to 500-m radius buffers evaluated from 2007 SPOT image and associated with the 1994-97 anopheline aggressiveness.

	2007 SPOT-5 image (2.5 m degraded to 20 m)			
	**Coefficient**	**95% IC**	**p-value**	**Adjusted R^2^**

Built-up area in 100-m radius buffer (per Ha)	-0.51	-0.73; -0.29	0.0001	0.49
				
Built-up area in 200-m radius buffer (per Ha)	-0.13	-0.18; -0.07	0.0001	0.51
				
Built-up area in 300-m radius buffer (per Ha)	-0.06	-0.07; -0.04	< 0.0001	0.57
				
Built-up area in 400-m radius buffer (per Ha)	-0.03	-0.04; -0.02	0.0001	0.53
				
Built-up area in 500-m radius buffer (per Ha)	-0.02	-0.02; -0.01	0.0004	0.44

Univariate linear regression. Twenty-three observations. Anopheline aggressiveness is quare root transformed.

Step 3. The GIS was used to extract the environmental values from the 2007 image at the 2007 catching point locations (Table 2). The Moran's I index was -0.07 with a z-score equal to 0.84 standard deviations. Those results were not statistically significant but in the absence of evidence of spatial autocorrelation between the 9 aggressiveness values from 2007, no cluster parameter was taken into account in the statistical analyses. Table 5 provides the parameters of the linear regression fitted for validation using the independent set of data (R^2 ^= 0.68).

**Table 5:** Validation of 2007 risk map with 2007 entomological figures.

	2007 SPOT-5 image (2.5 m degraded to 20 m) (Step 3)			
	**Coefficient**	**95% IC**	**p-value**	**Adjusted R^2^**

Built-up area in 300-m radius buffer (per Ha)	-0.07	-0.10; 0.03	0.0037	0.68

Univariate linear regression for nine observations.

Following the results of the Moran's I statistics at each of those 3 steps, no significant spatial autocorrelations could have been showed among the residuals of the fitted regressions.

### Risk maps

**Figure 1:**
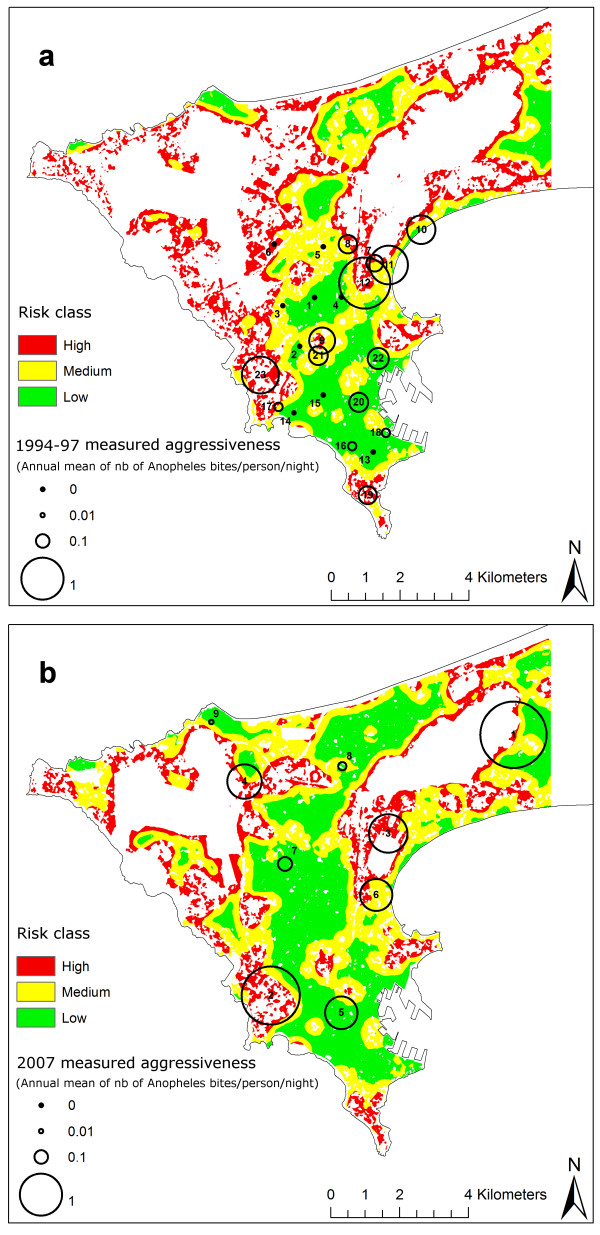
**Measured anopheline aggressiveness overlaid on risk maps computed using the built-up surface in a 300-m radius buffer for every pixel of the populated areas**. 1a. Anopheline data from 1994-97 and risk map computed with the 1996 SPOT satellite image. Refer to Table 1 for names of the study sites. 1b. Anopheline data from 2007 and risk map computed with the the 2007 SPOT satellite image. Refer to Table 2 for names of the study sites.

As the built-up area was found to be the factor most strongly associated with *Anopheles *density in the statistical analysis, risk maps were derived from the built-up area in a 300-m radius buffer for every pixel of the populated areas, using the results of the fitted regression model. Continuous values of the computed risk map were discretized to generate three classes, which were based on a calculation of the terciles of the built-up surface in the 300-m radius buffer, followed by a manual adjustment. Breaking values were chosen at 20 and 26 Ha around every pixel. Figures 1a and 1b show the 1996 and 2007 risk maps overlaid with the measured 1994-97 and 2007 *An. arabiensis *densities. A comparison of the maps indicated an increase in built-up surfaces in 11 years (about 1 300 ha), and also depicted the evolution of the areas of each risk class in Dakar and its suburb. Table 6 gives a summary of the information provided by the risk maps. The built-up area, the related anopheline aggressiveness predicted by inverting the best statistical association (R^2 ^= 0.57), the total surface of the risk class and the proportional surface of the risk class with respect to the total built-up area is given for each class. Table 1 and Table 2 provide the risk class at each catching point for the 1994-97 and 2007 studies, respectively.

**Table 6:** Description of the risk classes in 1996 and 2007.

Class	Risk	Built-up area in 300-m radius buffer	*An. arabiensis *aggressiveness evaluated from the model (number of bites/person/night)	Area of the risk class in 1996 (Ha)	Area of the risk class in 2007 (Ha)	Difference in risk class areas between 1996 and 2007	% difference in risk class areas between 1996 and 2007	% of the risk class area in 1996	% of the risk class area in 2007
1	High	< 20 ha	[2.82 - 0.28[	1457	1195	-262	-18%	32%	20%
2	Medium	[20-26[ ha	[0.28 - 0.03[	1735	2267	+532	+31%	39%	39%
3	Low	> = 26 ha	< = 0.03	1319	2385	+1066	+81%	29%	41%
									
Total				4511	5847	+1336	+30%	100%	100%

All values are extracted from Figure 1.

## Discussion

### Association between remotely sensed environmental data and *An. arabiensis *densities

Statistical associations were found between 1994-97 *Anopheles *aggressiveness [25,26] and 1996 and 2007 SPOT images. Even with the relatively small number of observations, aggressiveness values were statistically associated with the distance between the vegetation and the catching points and the built-up area in a 300-m radius buffer around the catching points. No multivariate model could be implemented because both ecological variables were highly correlated. Indeed, the presence of built-up areas was mainly colinear with the absence of vegetation. The validity of the model was assessed by fitting the model with an independent set of data. The aggressiveness recorded in 2007 was significantly associated with environmental data extracted from the 2007 image. The resulting agreement (R^2 ^= 0.68) improves our confidence in the statistical results from the present study.

The results of the present study provide evidence that environmental data retrieved from high spatial resolution SPOT satellite images (acquired for the rainy season) can be associated with *An. arabiensis *densities in the urban setting of Dakar. Almost 60% of the anopheline aggressiveness variability was explained with only one variable, the built-up area in a 300-m radius buffer, in a linear regression model.

### Risk maps

Comparable risk maps were drawn for 1996 and 2007 because the same ecological information could be extracted for both dates. Because the amount of built-up area in a 300-m radius buffer gave the best R^2 ^in the linear regressions, maps were computed based on this predictor. The built-up area in a 300-m buffer was calculated for every populated pixel of both images. Indeed, it is known that the peak of anopheline aggressiveness occurs in the middle of the night [14]. Because the evening and night activities are expected to take place mainly in or around dwellings, the non built-up areas were excluded from predictions.

### Evolution of urbanization

According to the report of the United Nations on population prospects' [1], 3 600 000 people lived in urban settings in Senegal in 1995, which grew to 4 890 000 in 2005, an increase of 36%. The results of the present study showed that urbanized areas in Dakar rose from 4510 Ha to 5847 Ha (+30%) between 1996 and 2007. Assuming that urban surfaces are proportional with the population figures and that the population increase in Dakar is proportional with the increase in the Senegalese population as a whole; the results are consistent with the increase in population. Most of the newly built-up areas were located around the airport located in the North-West of the city (in Almadies, north and west of CICES, Ouakam, Mermoz), near the Corniche (the sea coast west of the city), south of the Grande Niaye (in Hann Maristes) and around Yoff Plage (the long beach north of the city). The examination of the 1996 and 2007 maps confirmed that the city centre did not experience significant changes. Instead, the city is growing at its periphery where empty spaces still exist.

In Dakar, there was relatively little conversion from built-up zones to non-urban areas, which contrasts with Malindi and Kisumu, Kenya, where comparison of multi-date images showed important changes from urban to non-urban areas [23]. In Dakar, no major climatic or political events occurred, and desapearance of buildings can be related to destruction of slums or minor changes due to the 2006 flood.

### Malaria transmission risk evolution

The distribution of the risk classes evolved over time. The high-risk surface slightly decreased over 11 years (-262 Ha). Consequently, the raw number of at-high risk persons also slightly decreased. The geographical distribution of this class did not notably change. Indeed, the majority of the high-risk areas are located around the airport, the "Grande Niaye" (big marshland) and the University, which are places that have been quite stable over time. However, relative to the amount of built-up area in Dakar, the high-risk surface decreased significantly from 32 to 20%, and consequently, the proportion of the population at risk diminished between 1996 and 2007.

In contrast, the raw surface of the low-risk class increased greatly (+1066 Ha). In proportion of the total population also, the low-risk area rose significantly from 29 to 41%, meaning that both the raw number and the percentage of the urban population that is less exposed to malaria risk were greater in 2007 than 11 years before.

Finally, the raw surface of the medium-risk class increased moderately (+532 Ha), but the proportion of the population exposed to this medium transmission risk remained stable over the 11 years (39% of the total population).

These results highlight the benefits of urbanization in Dakar where the total population increased but the proportion of the population at higher risk for malaria transmission greatly decreased.

### Persistence of the associations

The persistence of the associations between *An. arabiensis *densities and ecological data was shown in the Dakar urban centre. Statistical results (*i.e*. estimated parameters) were similar when analysing the association between the 1994-97 agressiveness and the 1996 image in one hand, and analysing the association between the 2007 aggressiveness and the 2007 image in the other hand. Thus the relationship between the environment and the anopheles densities remained unchanged at a decadal time scale. No other parameters, such as a, evolution of antivectorial methods, were introduced in Dakar to modify this relationship. In addition, statistical associations remained significant when fitting the linear regression between the 1994-97 aggressiveness and the data issued from the 2007 image. Although one site changed (Bop - Cerf Volant, where slums were destroyed), the land cover of central and southern Dakar did not change significantly. This is consistent with the fact that Dakar is now evolving outside of its "historical" city centre. Thus, in city centres or places that are remaining stable, remotely sensed data could be used to predict vectorial risk even if only former and no contemporary data from the ground are available.

### Urbanization

Sparsely built-up areas are known to be risk factors for malaria in cities [24]. Furthermore, it is known that malaria transmission is reduced in urban centres compared to peri-urban and rural areas [2,3]. The present results confirm these patterns, as a highly built-up area around a catching point was a protective factor in the statistical model and was associated with lower *An. arabiensis *densities. Regarding scales of associations, aggressiveness was found to be associated with a 300-m radius buffer, which is consistent with previous findings in Pikine that found that most *An. arabiensis *were caught less than 285 m from the marshland, *i.e*. the breeding sites [11].

### Vegetation

In the present work, the NDVI has been used for vegetation mapping. It is a common index that quantifies coverage by green leaf vegetation [36] and captures some combined effects of temperature, humidity, rainfall, sunlight, altitude, land-use and land-cover in one value. The NDVI threshold was defined specifically for both the 1996 and 2007 satellite images to delineate the vegetation in the Dakar urban setting. Distance to vegetation was associated with *An. arabiensis *densities, which is consistent with several previous studies that suggested that vegetation, as measured with the NDVI, is a factor associated with the risk for malaria [37-40]. In the present study the NDVI has been used for the definition of the "vegetation areas" (*i.e*. presence/absence of vegetation) instead of the results of the supervised classification that distinguished several types of vegetations and associated bare soils (*i.e*. characteristics of vegetation). The small number of entomological observations did not allow any powered analysis of the association between the aggressiveness and the different classes of vegetation. Indeed, vegetation can play various roles in malaria transmission, depending on its characteristics. It can provide resting or feeding sites for mosquitoes or can be a proxy for the presence of breeding sites. For example, in Dakar, the "Grande Niaye" is a large, vegetated marshland known to provide habitat for mosquito breeding activity [11]. The presence of vegetation can also be an indicator of the presence of urban agriculture, which was reported to be associated with malaria in several African cities, such as in Côte d'Ivoire [41] and Ghana where irrigation led to the emergence of larval habitats [42,43] and a higher malaria prevalence [44,45]. In Dakar, non-cemented wells, locally called "ceanes", are used for market-garden activity and are known to be *Anopheles *breeding sites [14,46]. In addition, urban agriculture may provide potential resting sites for vectors [47]. It is also recognized that modifications in the vegetation cover, such as deforestation, are associated with changes in malaria transmission level [48]. Finally, vegetation type can be a determinant of mosquito density [49].

### Remote sensing in cities

In cities, there are obstacles to the use of remotely sensed data. Urban cover is spatially highly heterogeneous, the number of different building materials is high and the occurrence of mixed pixels is important [50]. Thus, even with high or very high spatial resolution images, distinguishing urban land uses and land covers could be difficult. Despite these difficulties, satellite images have been used in several ways in cities for a few years, and studies have attempted to describe vector presence and density or other malariometric indices.

The results of the present study are consistent with other findings in urban settings where malaria risk has been studied using environmental proxies of the presence of breeding sites and the distance to known breeding sites. In the cities of Malindi and Kisumu, Kenya, the NDVI was associated with a low housing density and thus with a higher probability of *Anopheles *breeding sites. The scale of the study was 270*270 m [22]. Using 15-m to 30-m resolution ASTER (Advanced Spaceborne Thermal Emission and Reflection Radiometer) images in New Haven, United States, the amount of vegetation in 50-m buffers around Aedes and Culex capture point, as well as the distance to water bodies, were related to mosquito densities [51].

As it has been done in this study, environmental changes and their impact on malaria risk have been studied with remotely sensed data. In two cities in Kenya, MTI (Multi-spectral Thermal Imager) satellite images acquired at 14-year intervals were used to detect changes in land use and land cover and showed that the presence, abundance and spatial distribution of breeding sites were driven by the evolution in the urbanization. Larval-positive water collections were primarily found in changing environments [23]. In Brazil, environmental changes due to the creation of a dam, as mapped from 1996 and 2001 Landsat-5 images were related to malaria incidence [52].

Other works have used different approaches, such as the study of other malariometric indices or the direct mapping of breeding sites. In Ouagadougou, Burkina Faso, overall prevalence of anti-CSP (circumsporozoite) antibodies and *P. falciparum *infections among children were associated with specific urban environments that were partly defined on a SPOT-5 satellite image [24]. In Malindi and Kisumu, Kenya, researchers attempted to directly identify *An. gambiae s.l*., *An. funestus *and *Anopheles merus *breeding sites using 5- to 20-m spatial resolution MTI images, but only 6% of the sites were detected [21]. On the contrary, in Dar-Es-Salaam, Tanzania, aerial photographs were visually interpreted to identify breeding sites and thus helped guide an integrated fight against malaria in the city [53].

Finally, radar images have been useful in the study of malaria in tropical areas [54,55], even in urban settings [56]. Indeed, they have a powerful capacity for detecting water and do not have cloud cover acquisition problems.

### Validity of image classification

The kappa coefficient calculated following the supervised classification step was 0.85 in 1996 and 0.95 in 2007, indicating a strong agreement between ground-truthed data and the classes from the supervised classification. Most of the confusions were not of major importance because they occurred mainly between different urban types, which were further aggregated in the built-up class. However, other confusions could not be totally erased, such as water and some types of asphalt.

### Comparison of multi-date images

Comparing images can be difficult because some differences are not due to actual ecological differences; instead, they are related to the specificity of the images, such as differences in atmospheric conditions, solar angle, sensor calibration, period of the view or the registration of images [57]. The method used in the present study improved the validity of the comparison in several ways. First, comparisons between images were not done directly on the images but rather on the results of classifications and calculations of the vegetation index, thus taking into account intrinsic parameters for each image. Furthermore, images were acquired in level 3 pre-processing, thereby avoiding all problems of spatial misregistration. Finally, differences in atmospheric conditions were taken into account thanks to the IARR pre-processing. This was of particular importance because it allowed an NDVI common threshold to be chosen for the 1996 and 2007 images, which was not feasible without this pre-processing step.

Comparison of environmental data from multi-date images was also made possible because views were acquired from the same period of the year (during the wet season). This was of major importance, especially for vegetation measurement. Rainfall was 350 mm in 1996 and 178 mm in 2007, but images were both taken just a few days after the last rain (13 days in 1996 and 10 days in 2007). At that time, the vegetation should have been at its maximum growth so vegetation developments were considered comparable.

The validity of the comparison of the supervised classifications in 1996 and 2007 was inescapably related to differences in spatial and spectral resolutions. The 1996 SPOT-4 image has three bands at 20 m whereas the 2007 SPOT-5 image had three bands at 2.5 m and one band at 10 m. Even if the 2007 image was resampled to 20 m to allow comparison with the 1996 image, every averaged 20-m pixel may contain more information than pixels initially aqcuired at 20-m spatial resolution. In Korea, it was shown that about 20% of a scene could have been classified into different classes based on two images at different spatial resolutions (Ikonos and Landsat) [58]. Whereas this could impede the validity of multi-date comparisons, choosing common training polygons for supervised classification of both images should have improved this quality.

## Conclusion

Remotely sensed environmental data were statistically associated with the *An. arabiensis *densities in Dakar city. Accordingly, risks maps were drawn for the years 1996 and 2007. Based on these maps, urbanization led to an increase in the proportion of the population at low risk for malaria transmission, *i.e*., when urbanization increased, malaria risk was reduced. These maps should be seen as a first step towards creating operational risk maps that could drive antivectorial control in the city.

## Competing interests

The authors declare that they have no competing interests.

## Authors' contributions

VM was responsible for study design, analysis, remotely sensed images processing, interpretation, production of the final manuscript and revisions. CV contributed to the study design, remotely sensed images processing, analysis, interpretation and production of the final manuscript and revisions. LG was co-responsible for field data collection. FP was responsible for overall scientific management, study design, analysis, interpretation, preparation of the final manuscript and revisions. CS contributed to overall scientific management. JFT contributed to overall scientific management. JPL was responsible for overall scientific management, study design, analysis, interpretation, preparation of the final manuscript and revisions. CR was responsible for overall scientific management, study design, analysis, interpretation, preparation of the final manuscript and revisions. All authors read and approved the final manuscript.

## Supplementary Material

Additional file 1 **Spatial repartition of the training polygons digitized for supervised classification process**. Click here for file
