# Supplementary figures and images for: Spatial heterogeneity and temporal evolution of malaria transmission risk in Dakar, Senegal, according to remotely sensed environmental data
Source: Malar J. 2010 Sep 3;9:252. doi: 10.1186/1475-2875-9-252 (PMC2944340; doi:10.1186/1475-2875-9-252)

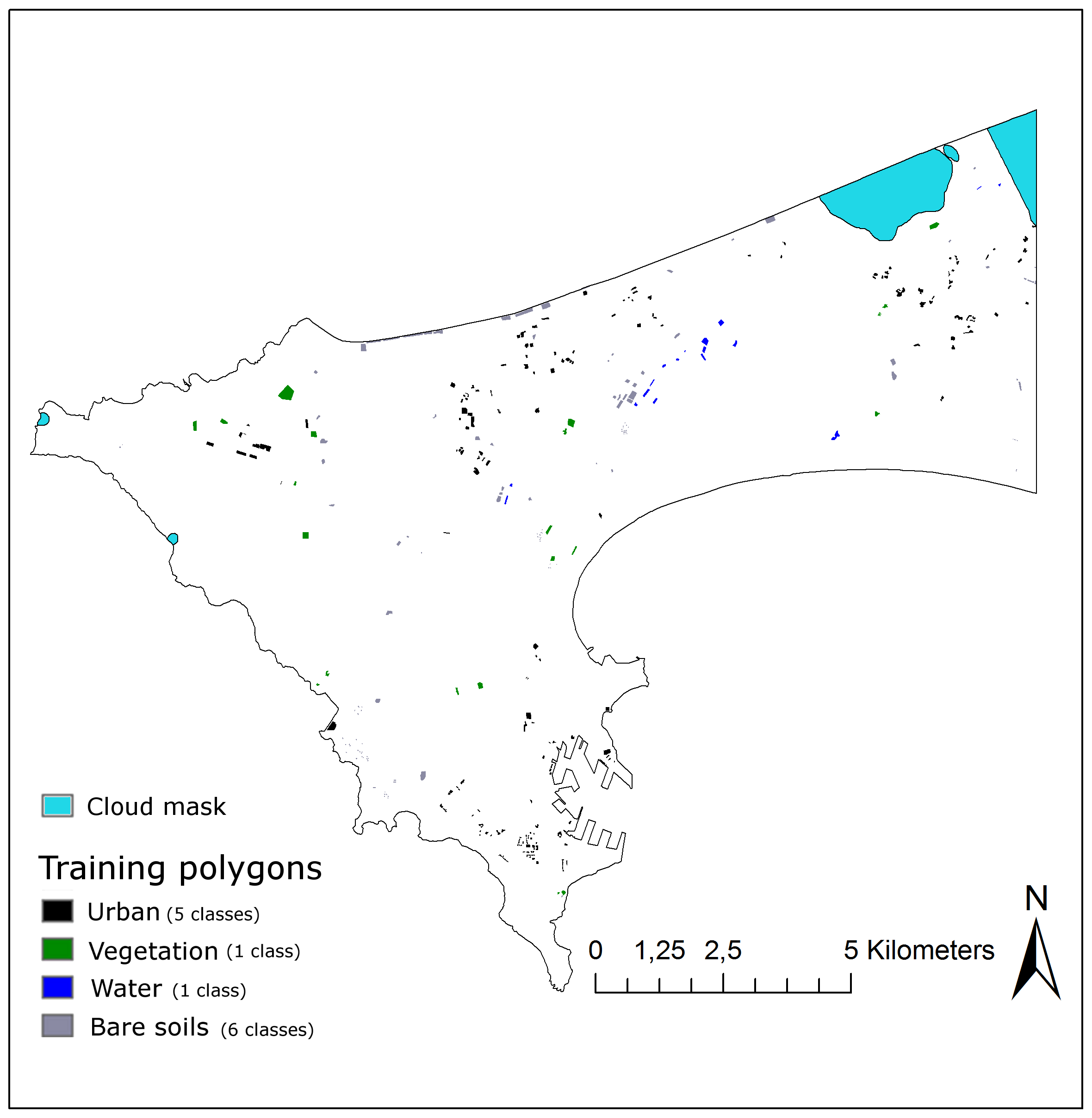

Supplement: Additional file 1 — Spatial repartition of the training polygons digitized for supervised classification process. [file 1475-2875-9-252-S1.PNG]
